# Titles of Papers, Addresses, Etc., Published by Dr. Roswell Park between 1881 and 1914

**Published:** 1914-03

**Authors:** 


### Titles of Papers, Addresses, Etc., Published by Dr. Roswell Park Between 1881 and 1914.

Besides the 155 titles included in this list there are some 25 published clinical lectures, with numerous articles in Johnson's Cyclopædia and Editorial articles in the *Annals of Surgery,* the *Weekly Medical Review,* the *Medical Press of* Western New York, the *Atlantic Medical Weekly,* and minor contributions in various journals.

1881.

1. On the Surgical Anatomy of the Sheaths of the Palmar Tendons. *Annals of Anatomy and Surgery.* August, 1881.

1882.

2. A Case of Severe Injury to the Orbit. *Archives of Opthalmology.* March, 1882.
3. On a New Method of Making Anatomical Preparations. *Annals of Anatomy and Surgery.* March, 1882.
4. The Present Status of Antiseptic Surgery. *Chicago Medical Journal and Examiner.* October, 1882.

1883.

5. Secondary Batteries and the Storage of Electricity. *Chicago Medical Journal and Examiner.* February, 1883.

6. The Electric Light in Diagnosis. *Annals of Anatomy and Surgery.* March, 1883.

7. Primary Antiseptic Occlusion of Gun-shot Wounds. Edit. in *Annals of Anatomy and Surgery.*

8. Record of Principal Anatomical Anomalies noticed during the Dissection of 100 Subjects. *Annals of Anatomy and Surgery.* December, 1883.

1884.

9. Tuberculosis of Bones and Joints; Treatment by Ignipuncture. *Philadelphia Medical News.* August 30th, 1884.

10. Report on Surgery to the Illinois State Medical Society. *Weekly Medical Review.* June, 1884.

11. Surgery of the Nerves. *Kansas City Medical Index.* April, 1884.

12. On Fat Embolism. *New York Medical Journal.* August 16th, 1884.

13. Select Topics in the Surgery of the Nervous System. *Weekly Medical Review.* May 17th, 1884.

14. Tuberculosis of Joints. *Weekly Medical Review.* April 28th, 1884.

1885.

15. The Conditions Which Predispose Bones to Tuberculosis. *Western New York Medical Press.*

16. Tuberculous Surgical Affections. Edit. in *Annals of Surgery.*

17. The Early Treatment of Gun-shot Wounds. *Physicians' Magazine.* August. 1885.

18. The Surgical Sequelæ of the Exanthems and Continued Fevers. *Canadian Practitioner.* July, 1885.

19. Antiseptics. Wood's *Reference Handbook.*

20. A Case of Total Extirpation of the Larynx. *Medical Press of Western New York.* December, 1885.

21. Tuberculosis of Glands, Etc. Edit. in *Annals of Surgery.*

1886.

22. Cystic Degeneration of Kidney; Nephrectomy on the Youngest Patient Ever Surving the Operation. *Medical Press of Western New York.* August, 1886. *Philadelphia Medical News.* May 22d, 1886.

23. Wounds of Head. Wood's *Reference Handbook.*

24. Lipoma Testis. Trans. *American Surgical Association.*

25. Electricity in Surgery. Wood's *Reference Handbook.*

26. Oesophageal Diverticulum. *Medical Press of Western New York.*

1887.

27. Address on Congenital Deformities of Mouth and Face. *Independent Practitioner* (Buffalo). November, 1887.

28. Intubation versus Tracheotomy. *Western New York Medical Press.* September, 1887.

29. A Further Study of Tuberculosis of Bone. *Western New York Medical Press.* January, 1887.

30. Laryngectomy. Wood's *Reference Handbook.*

1888.

31. Surgery of the Brain, based upon Principles of Cerebral Localization. Trans. American Congress Physicians and Surgeons. Volume 1. *New York Medical Journal.* November, 1888. (Three issues, pp. 63.)

32. A Study of Some of the Pyogenic Bacteria, Etc. Trans. American Surgical Association, 1888. *Philadelphia Medical News.* December 1st, 1888.

33. Extensive Thoracotomy for Sarcoma of Chest Wall. *Annals of Surgery,* 1888.

34. Abscess Containing Microccus Tetragenus. Trans. American Surgical Association, 1888.

35. Monograph, Diseases of the Breast Other Than Tumors. In Mann's *System of Gynecology.* Vol. II, pp. 34.

36. Splenectomy for Leucæmic Enlargement. *Annals of Surgery.*

37. Pylorus, Resection of. Wood's *Reference Handbook.*

38. Contributions to Abdominal Surgery. *Western New York Medical Press.* August, 1888.

39. Laparotomy for Gun-shot Wound of Abdomen. *Philadelphia Medical News.* August 4th, 1888.

40. Pyemia as a Direct Sequel of Gonorrhœa. *Journal of Cutaneous and Venereal Diseases.* December, 1888.

41. Laporotomy or Enterostomy. *New York Medical Record.* March 3d, 1888.

1889.

42. The Pathology of Suppuration. *Buffalo Medical Journal.*

43. A Study of Acute Infectious Processes in Bone. *American Journal of Medical Sciences.* June, 1889.

1890.

44. Monograph on the Congenital Defects and Deformities of the Face, Lips, Mouth, Tongue and Jaws. In Keating's *Cyclopedia of Diseases of Children.*

45. Art. "Trephining." Wood's *Reference Handbook.*

1891.

46. Wound Infection, Etc. *American Journal of Medical Sciences*. November, 1891.

47. A Study of Atrophy. Trans. American Orthopedic Association, 1891.

1892.

48. Clinical Contributions to the Subject of Brain Surgery. *Philadelphia Medical News*. December, 1892.

49. Monograph on Peritonitis, Appendicitis and Perityphilitic Abscess. In Hare's *Cyclopedia of Therapeutics* (20 pp.)

50. The Mutter Lectures on Surgical Pathology. Being a course of ten lectures delivered at the College of Physicians in Philadelphia, and published in the *Annals of Surgery*, Vols. 13, 14, 15. Published in book form, 300 pages, with above title, by J. H. Chambers & Co., St. Louis.

1893.

51. Actinomycosis, with Report of Case. *Buffalo Medical Journal*. January, 1893.

52. The Parasitic Theory of the Etiology of Carcinoma. *New York Medical Journal*. March, 4th, 1893. Trans. Medical Society State of New York. 1893.

53. Report of Case of Malignant Polyp from Base of Skull, Etc. *Annals of Surgery*. Trans. American Surgical Association, 1893.

54. The Importance to the Surgeon of Familiarity with the Bacillus Coli Communis Ibid.

1894.

55. On the Value to the Surgeon of Antipyrine, Etc. *Philadelphia Medical News*. December, 1894.

56. Forms of Peritonitis, Their Relation to Appendicitis, Etc. *Detroit Medical Age*. January 25th, 1894.

57. Rare Case of Fracture-Dislocation of the Vertebræ, With Recovery. *International Medical Magazine*. 1894.

1895.

58. A Series of Ten Lectures on the History of Medicine. Delivered at the University of Buffalo, Session of 1894-5. Published in the *Detroit Medical Age*. In book form, with much additional matter, by the F. A. Davis Co., Philadelphia. Two Editions.

59. The Location and Detection of Missiles. *Detroit Medicine*. June, 1895.

60. A Case of Spina Bifida Treated by Insertion of Celluloid Plate. *Buffalo Medical Journal.* August, 1895.

61. Fracture Into and Near Joints. *Atlantic Medical Weekly.* June 15th, 1895.

62. On the Consequences of Hyperemia and the Pathology of Inflammation, Etc. *New York Medical Record.* June 8th, 1895.

63. Acute Infectious Processes in Bone. *Boston Medical and Surgical Journal.* May 2d, 1895.

64. Results of the Division of the Pneumogastric and Phrenic Nerves, Etc. *Annals of Surgery.* August 1st, 1895.

65. A Case of Acromegaly. *International Medical Magazine.* July 1st, 1895.

66. Orchidomeningitis Calcificans. *Journal of Cutaneous and Venereal Diseases.* September, 1895.

67. On the Deformities and Malformations Resulting From Acute Infections in Bone. *New York Medical Record.* November 2d, 1895.

68. Applications of a Knowledge of Bacteriology to Certain Surgical Affections. *Atlantic Medical Weekly.* February 9th, 1895.

69. Septic Infection Within the Cranium. *Chicago Medical Record.* February, 1895.

70. Record of Foreign Bodies Found in the Appendix. *New York Medical Record.* March 16th, 1895.

71. Surgical Diseases and Injuries to the Head. Monograph of 300 pages in Dennis and Billings' System of American Surgery.

1896.

72. History of the Introduction of Anæsthesia Into Surgery. *Buffalo Medical Journal.* November, 1896.

73. On Auto-Infection, especially in Surgical Patients. *Chicago Corpuscle.* June, 1896.

74. Susceptibility and Immunity with special reference to Surgical Cases. Trans. American Surgical Association, 1896. Vol. XIV.

75. On Congenital Fistulæ and Sinuses at the Umbilicus. *St. Louis Medical Fortnightly.* January 1st, 1896. *St. Louis Medical Journal.* January, 1896.

76. A New Ideal Styptic. *New York Therapeutic Progress,* January, 1896.

77. History of Dentistry. *Buffalo Dental Practitioner.* January, 1896.

78. Pathology of Wolffian Remains. *International Journal of Surgery.* January, 1896.

79. On the Value of a Knowledge of Comparative Pathology to the Surgeon. President's Address before the Medical Society of the State of New York. *Medical News.* February, 1896.

80. Surgery by American Authors. Vols. II, 1896. Edited and largely written by Roswell Park. Lea Bros. & Co., Philadelphia, Pa.

81. Surgery by American Authors. 2d Edition, 1898. Same as above. 1 Volume.

82. Surgery by American Authors. 3d Edition, 1901. Same as above. 1 Volume.

1897.

83. An Epitome of an Address on Injuries to the Head. *Yale Medical Journal.* January, 1897.

1898.

84. An Inquiry Into the Etiology of Cancer. *American Journal of Medical Sciences.* May, 1898.

85. Indications for Operation in Tuberculosis of Kidney. *Journal of Cutaneous and Genito-Urinary Diseases.* August, 1898.

86. Iatro-Theurigic Symbolism. Maine Medical Association. June 2d, 1898.

87. Evolution of the Surgeon to the Barber. Janus. July, 1898.

88. Gun-shot Wounds Made by Modern Missiles and Their Treatment. *Buffalo Medical Journal.* August, 1898.

89. The New York State Cancer Laboratory at Buffalo. *Charlotte Medical Journal.* September, 1898.

1899.

Evolution of the Surgeon to the Barber. *Indian Lancet.* January 1st, 1899.

90. A Further Study Into the Frequency and Nature of Cancer. *Medical News.* April 1st, 1899.

91. Cancer as a Parasitic Disease. *Maryland Medical Journal.* June 17th, 1899.

92. History of Opo-or Organo-Therapy. *St. Louis Courier of Medicine.* July, 1899.

Evolution of the Surgeon From the Barber. Janus. August, 1899.

1900.

93. Again the Question of Cancer. *Buffalo Medical Journal.* March, 1900.

94. The Etiology of Cancer. *Medical News.* March 3d, 1900.

95. The Nature of the Cancerous Process and the Cancerous Cachexia and the Relation of Local Irritation to Each. *American Medical Quarterly*. April, 1900. (February 14th).

96. The Recent Buffalo Investigations Regarding the Nature of Cancer. Address to the American Medical Association. *American Medicine*. April 13th, 1901. Vol 1, page 65.

96B. Doctorate Address, Kentucky School of Medicine, June, 1900. *American Practitioner and News*. July 15, 1900, p 41.

1901.

97. The Nature of the Cancerous Process. *Journal of American Medical Association*. September 14th, 1901.

1902.

98. Surgical Treatment of Injuries and Diseases of the Pancreas. *American Medicine*. February 15th, 1902. *Journal of Medicine and Surgery*, Savannah, Ga. March, 1902.

Surgical Treatment of Injuries and Diseases of the Pancreas. Printed in Japan. May, 1902.

99. Head. Wounds of. *Reference Handbook of Medical Science*. April, 1902.

100. Student Life in the Middle Ages. *University of Penna. Medical Bulletin*. March, 1902.

101. The Present Aspect of the Cancer Problem. *Medical Review of Reviews*. June, 1902.

102. The Advantages of Early Surgical Intervention in Borderland Cases. *Medical News*, New York. June 7th, 1902.

103. A Study of Medical Words, Deeds and Men. Address delivered June 24th, 1902, at Yale University Commencement. *Yale Medical Journal*. July, 1902.

104. Why Should We Not Treat the Gall Bladder as We do the Appendix. *American Medicine*. July 12th, 1902. (Vol. 4, No. 2, page 54).

105. Surgical Treatment of Epilepsy. *American Medicine*. November 5th, 1902.

1903.

Trans. of the National Association for the Study of Epilepsy. Volume 2, November, 1903.

106. Sarcoma of the Third Cervical Segment; Operation; Removal; Continued Improvement. *American Journal of Medical Sciences*. January, 1903.

107. The Modern Therapy of Septic Puerperal Infection. *Alpha Omega Delta Bulletin*. March, 1903. *New England Medical Monthly*. May, 1903.

108. Successful Removal of 265 cm. of Gangrenous Intestine. *Buffalo Medical Journal.* April, 1903. *International Journal of Surgery.*. Vol. 1, No. 1.

109. Report upon the Physic and Therapeutic Value of Cathode and Ultra-Violet Rays: With Reference to the Electromagnetic Theory of Light; an attempt to more clearly define the general character of these recently introduced agents in the treatment of Cancer. *Medical News,* New York. May 30th, 1903.

110. On Cysts and Other Neoplasms of the Pancreas. *American Medicine.* Vol. 5, No. 24, pages 949-954. June 13th, 1903.

111. An Epitome of the History of Carcinoma. *Medical Library and Historical Journal.* October, 1903. *Johns Hopkins Bulletin.* October, 1903.

112. Successful Removal of 265 cm. of Gangrenous Intestine. *Archives Internationales de Chirurgie.* 1903.

1904.

113. Histoire de la Medecine en Amerique. *La France Medicale.* February 10th, 1904.

114. Surgery in America. *Encyclopedia Americana.* Volume XV. March, 1904.

115. Spontaneous Gangrene of the Hollow Viscera. *Annals of Surgery.* April, 1904.

116. A Letter from Berlin. *Buffalo Medical Journal.* April 10th, 1904.

117. Surgical Treatment of Dyspepsia. *Buffalo Medical Journal.* July, 1904.

1905.

118. Contributions to the Literature of Foreign Bodies in the Pharynx and Oesophagus. *Buffalo Medical Journal.* Clinical Reports.

119. Bilateral Cervical Sympathectomy for the Relief of Epilepsy, with Reports of Three Cases; Notes on the Physiologic Effects of Cutting the Sympathetic, and on the Histologic Changes found in the Cases in Question. *Journal of Nervous and Mental Diseases.* April, 1905.

120. Address at the Commencement of the Buffalo General Hospital Training School. June 19th, 1905. *Dietetic and Hygenic Gazette.*

121. Status Lymphaticus and the Ductless Glands. Surgery, Gynecology and Obstetrics. August, 1905.

122. Oration on Surgery. *New York State Journal of Medicine.* March and April, 1906.

123. The Story of the Discovery of the Circulation. *Buffalo Medical Journal.* August, 1906.

124. A New Method of the Utilization of the Sac in the Radical Cure of Hernia. Surgery, Gynecology and Obstetrics. August, 1906.

125. What do Recent Studies Regarding the Thyroid and Parathyroids Teach Concerning the Treatment of Exophthalmic Goitre? *Medical Review of Reviews.* July and August, 1906.

1907.

126. The Work of the New York State Cancer Laboratory—Retrospective—Prospective. *New York State Journal of Medicine.* May, 1907.

127. Non-Inflammatory Affections of Bones. Volume 3, *American Practice of Surgery.* By Bryant and Buck.

128. The Modern Practice of Surgery. (1100 pages). Lea Bros., Philadelphia, Penna.

129. The Medico-Legal Consideration of Gun-shot Wounds. From 2d Edition of Withaus and Becker's *Medical Jurisprudence.* Revised by Author in 1893-4. Not published till 1906-7.

130. A Case of Cyst Within the Spinal Canal. By William Krauss, M. D., and Roswell Park, M. D. Brain. *Journal of Neurology.* 1907.

1908.

131. Some of the Modern Aspects of the Cancer Problem. Hartford Academy of Medicine. March 23d, 1908.

132. The Nature of the Cancerous Process. International Society of Surgery. Brussels, Belgium. September 21st, 1908. Published in Transactions of International Society.

133. Traitement du Cancer des Differents Organes. November 25th, 1908.

1909.

Introduction to "The Doctor in Art." Published by the Douglas Publishing Co., Buffalo, 1909.

135. The Career of the Military Surgeon. *Military Surgeon.* May 29th, 1909.

136. The Next Twenty-Five Years in Surgery. *Buffalo Medical Journal.* June, 1909.

137. The Relation of the Internal Secretions to Surgical Conditions. *Northwest Medicine,* Seattle. August, 1909.

1910.

138. The Cancer Problem. *Southern Medical Journal.* March, 1910.

139. Brewer's Yeast in the Treatment of Ulcers, Necrotic and Tubercular Conditions. *American Journal of Dermatology.* January, 1911.

1911.

140. Recent Views Concerning the Treatment of Cancer, Based Upon Its Nature. *Buffalo Medical Journal.* April, 1911.

141. Remarks on the Early History of Medicine in America. *Buffalo Medical Journal.* July, 1911.

1912.

142. Anæsthesia by Intra-Tracheal Insufflation. *Buffalo Medical Journal.* January, 1912.

143. Surgery of the Liver and Gallbladder. *Handbook of Practical Treatment.* Edited by Drs. Musser & Kelly, 1912.

144. If Washington Should Return to His Country. (Unpublished). University Day Address. February 22d, 1912.

145. Thanatology: A Questionaire and a Plea for a Neglected Study. *Journal American Medical Association.* April 27th, 1912.

146. The History of the Dentist's Art. *Dental Forum.* September, 1912.

147. The What and Why of Eugenics. *Buffalo Sunday News.* October 20th, 1912.

148. Isopathy in the Twentieth Century. *Post-Graduate.* October, 1912.

1913.

149. The Evil Eye, Thanatology and Other Essays. (380 pages). Boston: R. G. Badger, Publisher. January, 1913

150. Fracture of the Atlas: Separation of a Fragment and its Subsequent Extrusion Through the Mouth. (Case of Dr. James P. White). *Buffalo Medical Journal.* January, 1913.

151. Report of Fourteen Cases of Spina Bifida, and One of Sacrococcygeal Tumor. *Buffalo Medical Journal.* March, 1913.

152. What Prospect for a Successful Therapy of Cancer? *International Medical Journal.* May, 1913.

153. The Thymus and Other Ductless Glands. *Cleveland Medical Journal.* May, 1913.

154. Conclusions Drawn from a Quarter-Century's Work in Brain Surgery. *N. Y. State Journal of Medicine.* June, 1913.

1914.

155. On the Relation of the Ductless Glands to the Work of the Surgeon. (Read at Fourth Clinical Congress of Surgeons of North America. Chicago, November 14th, 1913). *Surgery, Gynecology and Obstetics,* March, 1914.

156. Brain Surgery. *Reference Handbook of Medical Sciences.*

157. Lecture. University of Buffalo. Radium and Radioactivity.

158. Of What Does the University Consist? *Buffalo Medical Journal.* March, 1914.

---

Dr. Frank Byron Brooks, Syracuse, 1881, died in Syracuse, December 26, aged 58.

---

Dr. Diogenes D. Case, Bellevue, 1869, died at his home in Morrisville, N. Y., January 7, aged 70.

---

Dr. Frank Knapp of Binghamton (not listed in State Directory), died January 12, aged 55.

---

Dr. Frederick E. Barrows of Utica (not listed in State Directory), died January 19, aged 64 years.

---

Dr. Ray Vaughan Pierce, Eclectic Medical Institute of Cincinnati, 1865, of Buffalo, died at his winter home, St. Vincent's Island, Fla., February 3, of cerebral hæmorrhage, aged 64.

---

Dr. Henry E. Zielly, Geneva Medical College, 1849, died at Spokane, Wash., November 18, 1913, aged 87.

---

NOTE.—Mention of the fact that a man is not listed in the State Directory, corrections of address, etc., are not intended in any sense as criticisms of the committees which have prepared this work. Eerrors in so extensive a work are inevitable and changes are frequent. Retired physicians seldom take the pains to keep their names in the special list published. Again, especially in death notices, we cannot be sure that all doctors are doctors of medicine, hence we note the fact of absence from the State list, in order to facilitate corrections.

